# The associations of total testosterone with probable nonalcoholic steatohepatitis and nonalcoholic fatty liver disease fibrotic progression in men with type 2 diabetes: a cross-sectional study

**DOI:** 10.1186/s40001-022-00958-8

**Published:** 2022-12-27

**Authors:** Ziteng Zhang, Chi Chen, Yuying Wang, Ningjian Wang, Yi Chen, Yingli Lu, Fangzhen Xia

**Affiliations:** Institute and Department of Endocrinology and Metabolism, Shanghai Ninth People’s Hospital, Shanghai JiaoTong University School of Medicine, Zhizaoju Road No. 639, Shanghai, 200011 China

**Keywords:** Testosterone, Nonalcoholic fatty liver disease, Advanced fibrosis, Type 2 diabetes mellitus

## Abstract

**Background:**

Testosterone has an impact on metabolic disorders and men with type 2 diabetes mellitus (T2DM) are predisposed to hypogonadism; meanwhile, patients with T2DM have higher risk of NAFLD. Therefore, we speculate that testosterone may affect the progression of NAFLD in T2DM patients and we aim to investigate whether total testosterone is associated with NAFLD progression in men with T2DM.

**Methods:**

A cross-sectional study. A total of 1782 male participants with T2DM were enrolled from seven communities in Shanghai. Probable nonalcoholic steatohepatitis (NASH) was defined by the concurrence of NAFLD and metabolic syndrome (MetS). NAFLD fibrosis score was used to identify patients with probable advanced fibrosis. Multinomial logistic regression and ordinal logistic regression was used to measure the association of total testosterone (independent variable) and the progression category of NAFLD (dependent variable).

**Results:**

In male, TT quartiles were negatively associated with probable NASH (Q1 vs. Q4 OR 2.07 95% CI 1.31–3.28, *P* for trend = 0.001) and inflammatory progression of NAFLD with OR of 1 SD increment of ln (TT) 0.81 (95% CI 0.72–0.92, *P* for trend < 0.001), but positively with fibrotic progression (Q1 vs. Q4 OR 0.45, 95% CI 0.29–0.72, *P* for trend = 0.001) with OR of 1 SD increment of ln (TT) 1.24 (95% CI 1.07–1.45). According to stratified analyses, for inflammatory progression, the interactions of age strata, duration of diabetes strata, and dyslipidemia status with 1 SD increment of ln (TT) were significant (*P* for interaction 0.007, 0.003, and 0.012, respectively); as for fibrotic progression, we found no interactions (all *P* for interaction ≥ 0.05).

**Conclusions:**

Different associations between TT and inflammatory and fibrotic progression of NAFLD in male were observed, suggesting different roles of TT in inflammatory and fibrotic stages of NAFLD.

## Background

Nonalcoholic fatty liver disease (NAFLD) has become the most prevalent chronic liver disease not only in western countries but also in China [1, 2]. Patients with T2DM have higher risk of NAFLD [3]. Meanwhile, T2DM may promote the progression of NAFLD to nonalcoholic steatohepatitis (NASH), advanced fibrosis, and cirrhosis [3, 4]. NASH patients are at higher risk of advanced fibrosis [5] and fibrosis is one of the direct risk factors for overall mortality in patients with NAFLD [6]. So, more attention ought to be paid to the progression of NAFLD in diabetic patients.

Sex steroids have direct impact on both hepatic and systematic metabolism, thereby involving NAFLD pathobiology. Meanwhile, as an important organ which regulates glucolipid and sex steroid metabolism, liver has interaction effects with reproductive system, thus taking on sexual dimorphism [7]. It has been reported that relative to female, male sex is an independent risk factor of NAFLD [8, 9]. A cross-sectional study of 541 NASH patients reported that men are predisposed to advanced stage of NAFLD compared with premenopausal women [10]. Testosterone may account for this gender difference. Testosterone, usually considered as the main sex steroids in male, has gender-dimorphic impact on the development of metabolic disease, such as type 2 diabetes mellitus [11, 12], and in turn, men with T2DM are particularly susceptible to sex hormone disturbance or hypogonadism [13, 14]. What is more, preclinical studies demonstrated that testosterone can influence hepatic insulin sensitivity and lipid metabolism [15–17]. Although several studies have found the associations between low testosterone and NAFLD in men [18, 19], quite limited studies have demonstrated the association between TT and advanced NAFLD status, including NASH and advanced fibrosis in men with T2DM.

Thus, we assume that total testosterone may be associated with inflammatory and fibrotic progression of NAFLD in men with type 2 diabetes. Liver biopsies are unavailable in large epidemiology studies. Since MetS is one of the strong noninvasive NASH predictors and NASH risk increases with the increasing number of MetS components, we used MetS to define probable NASH [20]. In order to further identify liver fibrosis in patients with NAFLD we used NFS, a noninvasive system which can classify NAFLD patients as with or without advanced fibrosis [21].

Hence, in this large community-based study, we investigated the relationship between total testosterone and inflammatory and fibrotic progression of NAFLD assessed by aforementioned noninvasive methods in Chinese men with T2DM.

## Methods

### Study design and participants

**Fig. 1:**
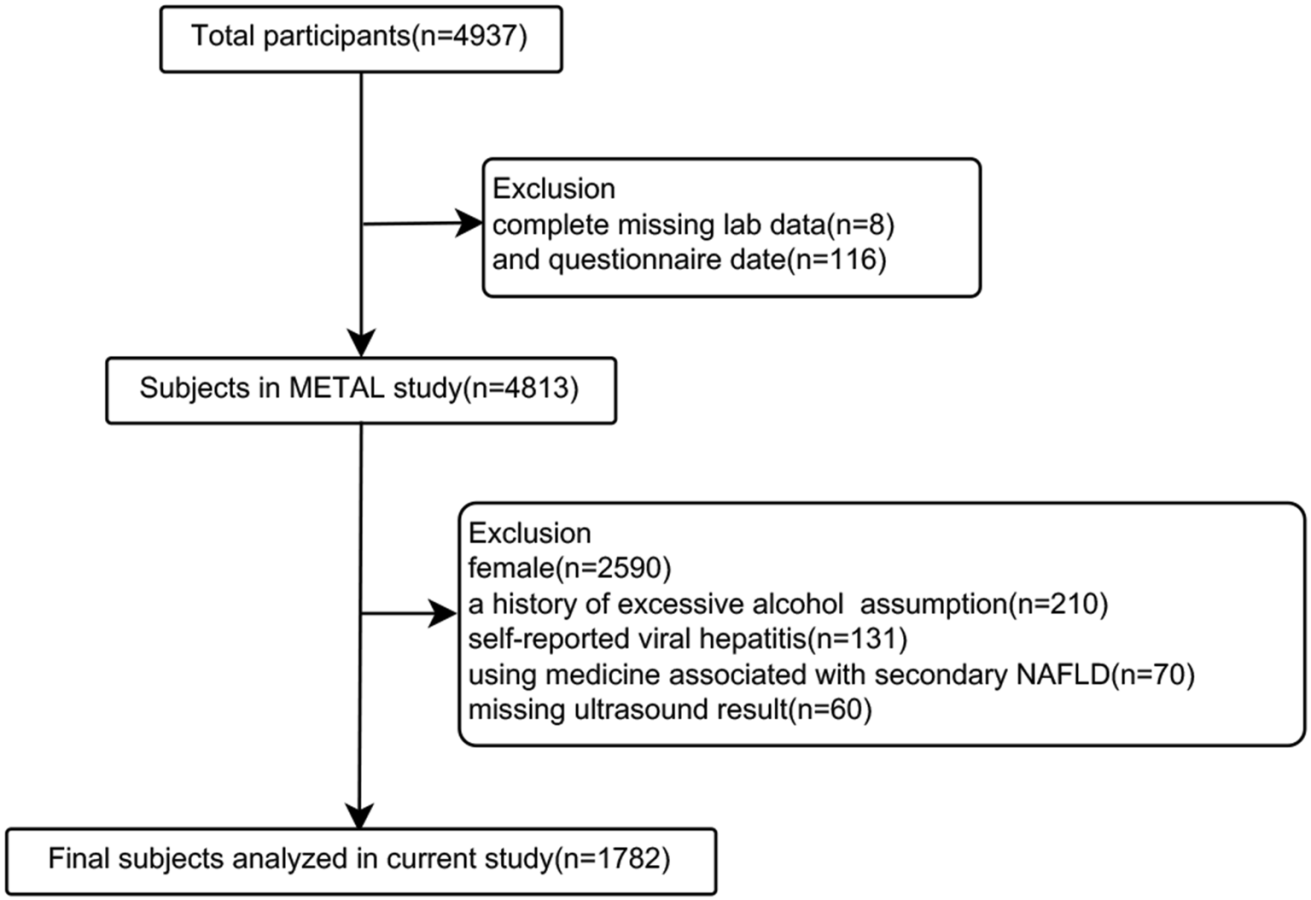
Flowchart of participants’ inclusion and exclusion

In 2018, we designed a population-based study named the METAL study (Environmental Pollutant Exposure and Metabolic Diseases in Shanghai, www.chictr.org.cn, ChiCTR1800017573). Participants from seven communities in Huangpu and Pudong District, Shanghai, China were enrolled in this study. In each community healthcare center, we used simple random sampling to select half of the diabetic patients from the registration platform. Chinese citizens ≥18 years old who had lived in their current area for ≥6 months were included. A total of 4937 subjects with diabetes received the examination. Participants who were missing laboratory results (*n* = 8) or questionnaire data (*n* = 116) were excluded. Further, female participants (*n* = 2590), who had a history of excessive consumption (male ≥ 140 g/week, female ≥ 70 g/week) of pure alcohol (Chinese Society of Hepatology 2010; *n* = 210), had self-reported viral hepatitis (including hepatitis B and hepatitis C virus; *n* = 131), were using medications associated with secondary NAFLD (corticosteroids, estrogens, amiodarone, methotrexate; *n* = 70), and did not have ultrasound results (*n* = 30), were excluded. Totally, 1782 male diabetic participants were involved in the final analyses (Fig. 1).

The study protocol was approved by the Ethics Committee of Shanghai Ninth People's Hospital, Shanghai Jiao Tong University School of Medicine. The study protocol conformed to the ethical guidelines of the Declaration of Helsinki as reflected in the a priori approval by the appropriate institutional review committee. Informed consent was obtained from all participants included in this study.

### Measurement

We followed the methods of Wang et al. [22] A questionnaire, including sociodemographic characteristics, family history, medical history, and lifestyle factors, was used to collect relevant data. The trained and experienced personnel were the same group from SPECT-China study [23]. The clinical examinations and interviews were conducted according to a standard protocol. Current smoking was defined as having smoked at least 100 cigarettes in one’s lifetime and currently smoking cigarettes [24]. Waist circumference was measured in the horizontal plane midway between the lowest ribs and the iliac crest at the end of a normal expiration, as suggested by the WHO and the International Diabetes Federation.

Blood samples were obtained between 6:00 a.m. and 9:00 a.m. after overnight fasting for at least 8 h. Blood was refrigerated immediately after phlebotomy and it was centrifugated within 2 h; the serum was aliquoted and frozen in a central laboratory. Total testosterone was detected with chemiluminescence method (Abbott i2000 SR, Chicago, USA) and the minimal detectable limit for it was 0.087 nmol/L. Glycated hemoglobin (HbA1c) was measured by HPLC (MQ-2000PT, Medconn, Shanghai, China). Fasting plasma glucose, alanine aminotransferase (ALT), aspartate aminotransferase (AST), and lipid profile were performed with a Beckman Coulter AU 680 (Brea, USA). Samples with values below the minimal detectable limit were assigned a value midway between zero and the minimal detectable limit for the analyses [25]. The inter- and intra-assay coefficients of variation were 8.33 and 6.25% for TT.

Hypertension was defined as systolic blood pressure ≥ 140 mmHg, diastolic blood pressure ≥ 90 mmHg, or self-reported previous diagnosis of hypertension by physicians. According to the modified National Cholesterol Education Program-Adult Treatment Panel III, dyslipidemia was defined as total cholesterol ≥ 6.22 mmol/L (240 mg/dL), triglycerides ≥ 2.26 mmol/L (200 mg/dL), low-density lipoprotein ≥ 4.14 mmol/L (160 mg/dL), high-density lipoprotein <1.04 mmol/L (40 mg/dL), or self-reported previous diagnosis of hyperlipidemia by physicians.

### Outcome definition

Liver fat accumulation (steatosis) was detected by ultrasound (Mindray M7, MINDRAY, Shenzhen, China) and the same sonologist [26, 27]. In accordance with Saadeh et al., presentation of steatosis included increased liver echogenicity, stronger echoes in the hepatic parenchyma compared to the renal parenchyma, vessel blurring, and narrowing of the lumen of the hepatic veins [28]. In patients with NAFLD, the presence of MetS is a strong predictor for nonalcoholic steatohepatitis (NASH) according to the guidelines by the American Association for the study of liver disease and the Chinese Society of Hepatology [29]. Thus, subjects with MetS and NAFLD were categorized as subjects with probable NASH and the rest were defined as simple NAFLD. The diagnosis of MetS is based on the International Diabetes Federation criteria (2005) [30]. A person with MetS must have the following criteria: abdominal obesity (waist circumference: male ≥ 90 cm, female ≥ 80 cm, or body mass index (BMI) is ≥30 kg/m2) plus any two of the following four parameters: (a) raised triglycerides ≥1.7 mmol/L, or treatment for this dyslipidemia; (b) reduced high‐density lipoprotein <1.03 mmol/L in men or <1.29 mmol/L in women, or treatment for this dyslipidemia; (c) raised blood pressure––systolic blood pressure ≥ 130 or diastolic blood pressure ≥ 85 mmHg, or treatment of hypertension; and (d) raised fasting plasma glucose ≥ 5.6 mmol/L or a history of type 2 diabetes.

The NFS was used to classified NAFLD patients into different risk groups of having advanced fibrosis [29]. The formula to calculate NFS is − 1.675 + 0.037*age (years) + 0.094*BMI (kg/m^2^) + 1.13*impaired fasting glucose/diabetes (yes = 1, no = 0) + 0.99*ST/ALT ratio − 0.013*platelet (*10^9^/L) − 0.66*albumin (g/dL) [21]. A score <− 1.455 was regarded as probable absence of advanced fibrosis with 90% sensitivity and 60% specificity to exclude advanced fibrosis; a score >0.676 was regarded as probable presence of advanced fibrosis with 67% sensitivity and 97% specificity to identify the presence of advanced fibrosis; and a score between − 1.455 and 0.676 has indeterminate results [21].

### Statistical analysis

Data analyses were performed using IBM SPSS Statistics, Version 26 (IBM Corporation, Armonk, New York, USA). Continuous variables are shown as mean ± SD while categorical variables as percentages (%). A *p* value <0.05 indicated significance (two-sided). The concentration of total testosterone was logarithmically transformed to achieve a normal distribution if necessary.

Total testosterone was divided into quartiles. The first quartile of total testosterone (Q1) represents the lowest one and the fourth quartile (Q4) represents the highest. Multinomial logistic regression was used to measure the association of testosterone (independent variable) and the progression category of NAFLD (dependent variable). Odds ratios (ORs) with 95% confidence interval (CI) were calculated by ordinal logistic regression to measure the association between the quartiles of testosterone, NAFLD inflammatory progression, and fibrotic progression. The model was adjusted for age, duration of diabetes, current smoking, waist circumference, dyslipidemia, and hypertension.

In subgroup analyses, logistic regression was used to explore the relationship between 1-SD increment of ln (TT) and inflammatory and fibrotic progression of NAFLD stratified by age (≥60 years or <60 years), waist circumference (≥90 cm or <90 cm), duration of diabetes (≥10 years or <10 years), hypertension (yes or no), and dyslipidemia (yes or no) and to examine the potential interaction association.

## Results

### Characteristics of the diabetic men by quartiles of TT

General clinical and demographic characteristics of the study population by quartiles of TT are shown in Table 1. The quartile ranges of TT were Q1 (≤10.75 nmol/L), Q2 (10.76–14.06 nmol/L), Q3 (14.07–17.88 nmol/L), and Q4 (≥17.89 nmol/L). With the elevation of testosterone quartiles, male subjects were older and had higher AST/ALT and HDL-C, lower morbidity of hypertension and dyslipidemia, platelet count, albumin, BMI, waist circumference, FPG, blood pressure and triglycerides, and a longer duration of diabetes. Meanwhile, the prevalence of probable NASH showed a significantly decreased trend; rather, increased yet not significant trend was observed in the prevalence of probable advanced fibrosis and NFS in men (Table 1).

**Table 1:** Characteristic of study population by quartiles of total testosterone (men, *n* = 1782)

Characteristic	Total testosterone (nmol/L)				*P* for trend
	Q1 (≤10.75)	Q2 (10.76–14.06)	Q3 (14.07–17.88)	Q4 (≥17.89)	
Men					
*N*	445	447	447	443	
Age, year	67.94 ± 9.64	67.16 ± 9.07	67.66 ± 8.13	68.93 ± 8.17	0.043
Duration of diabetes, year	9.64 ± 8.05	10.35 ± 7.89	9.80 ± 7.16	11.87 ± 8.50	<0.001
Current smoke, %	30.2	35.8	35.3	32.3	0.683
Platelet count, *10^9^/L	202.94 ± 52.40	204.43 ± 55.97	199.82 ± 54.54	195.59 ± 53.93	0.018
Albumin, g/dl	45.02 ± 2.59	44.81 ± 2.64	44.53 ± 2.68	44.24 ± 2.98	<0.001
AST/ALT	1.07 ± 0.41	1.09 ± 0.38	1.12 ± 0.35	1.22 ± 0.49	<0.001
BMI, kg/m^2^	26.25 ± 3.39	25.68 ± 3.36	24.72 ± 2.80	23.36 ± 2.93	<0.001
Waist circumference, cm	96.10 ± 8.93	94.05 ± 9.38	91.53 ± 7.62	87.41 ± 8.24	<0.001
FPG, mmol/L	8.20 ± 2.51	7.84 ± 2.46	7.65 ± 2.16	7.64 ± 2.21	<0.001
HbA1c, %	7.73 ± 1.38	7.65 ± 1.40	7.55 ± 1.39	7.57 ± 1.54	0.062
SBP, mmHg	146.74 ± 19.44	144.99 ± 18.91	142.99 ± 18.92	141.65 ± 19.64	<0.001
DBP, mmHg	80.73 ± 11.60	80.40 ± 10.89	80.44 ± 11.25	77.86 ± 10.22	<0.001
Total cholesterol, mmol/L	4.80 ± 1.19	4.72 ± 1.06	4.77 ± 1.03	4.90 ± 1.03	0.084
Triglycerides, mmol/L	2.29 ± 2.00	1.87 ± 1.42	1.70 ± 1.03	1.42 ± 0.92	<0.001
HDL-C, mmol/L	1.05 ± 0.22	1.06 ± 0.22	1.09 ± 0.23	1.22 ± 0.29	<0.001
LDL-C, mmol/L	2.96 ± 0.83	2.96 ± 0.78	2.99 ± 0.77	3.05 ± 0.76	0.068
Hypertension, %	84.7	80.1	77.4	73.1	<0.001
Dyslipidemia, %	72.1	71.8	64.2	49.0	<0.001
Simple NAFLD, %	16.6	19.2	20.4	19.6	<0.001
Probable NASH, %	67.4	55.3	45.2	28.2	<0.001
NFS	− 0.13 ± 0.99	− 0.18 ± 1.05	− 0.13 ± 0.96	− 0.06 ± 1.04	0.200
Probable NAFLD fibrosis, %	15.8	19.1	20.3	24.4	0.161

The data are summarized as the mean ± SD for continuous variables or as a percentage for categorical variables. P for trend was calculated by regression analysis. ALT, alanine aminotransferase; AST, aspartate aminotransferase; DBP, diastolic blood pressure; SBP, systolic blood pressure; FPG, fasting plasma glucose; HbA1c, glycated hemoglobin; HDL-C, high-density lipoprotein; LDL-C, low-density lipoprotein; NAFLD, nonalcoholic fatty liver disease; NASH, nonalcoholic steatohepatitis; NFS, NAFLD fibrosis score

### Association of TT with NAFLD inflammatory progression in diabetic men

Table 2 shows that TT of male diabetic patients was negatively associated with inflammatory progression of liver steatosis. In male, TT quartiles were negatively associated with simple NAFLD (Q1 vs. Q4 OR 2.73, 95% CI 1.70–4.39, *P* for trend <0.001) and probable NASH (Q1 vs. Q4 OR 2.07 95% CI 1.31–3.28, *P* for trend = 0.002) after adjustment for age, duration of diabetes, waist circumference, current smoking, hypertension, and dyslipidemia, and in ordinal regression, TT was also negatively associated with the inflammatory progression of NAFLD, with OR of 1 SD increment of ln (TT) 0.81 (95% CI 0.72–0.92, *P* for trend < 0.001).

**Table 2:** The association of testosterone with inflammatory progression of NAFLD

	Total testosterone^a^				*P* for trend	1 SD increment for ln (TT)
	Quartile 1	Quartile 2	Quartile 3	Quartile 4		
Men						
Simple NAFLD	2.73 (1.70–4.39)*	1.95 (1.27–3.00)*	1.54 (1.03–2.30)*	Ref.	<0.001	0.73 (0.62–0.86)*
Probable NASH	2.07 (1.31–3.28)*	1.22 (0.78–1.90)	1.05 (0.68–1.61)	Ref.	0.002	0.76 (0.64–0.91)*
Inflammatory of progression of NAFLD	1.83 (1.31–2.54)*	1.36 (0.99–1.87)	1.13 (0.84–1.54)	Ref.	<0.001	0.81 (0.72–0.92)*

Data are shown as regression coefficients or odds ratios (95% CI). Multinomial and ordinal logistic regression analyses were used

^a^Quartiles of total testosterone, nmol/L: men: ≤10.75, 10.76–14.06, 14.07–17.88, ≥17.89. Inflammatory progression of NAFLD, ranging from non-NAFLD to simple NAFLD and probable NASH; NAFLD, nonalcoholic fatty liver disease; NASH, nonalcoholic steatohepatitis. The model was adjusted for age, duration of diabetes, current smoking, waist circumference, dyslipidemia, and hypertension. *P < 0.05

### Association of TT with NAFLD fibrotic progression in diabetic men

Table 3 shows that TT of male was positively associated with fibrotic progression of liver steatosis. In men, TT quartiles were not associated with indeterminate group (*P* for trend = 0.697); however, unlike their negative association with the inflammatory progression, TT quartiles were positively associated with the fibrotic progression (Q1 vs. Q4 OR 0.45, 95% CI 0.29–0.72, *P* for trend = 0.001) after adjustment for age, duration of diabetes, waist circumference, current smoking, hypertension, and dyslipidemia, with OR of 1 SD increment of ln (TT) 1.24 (95% CI 1.07–1.45).

**Table 3:** The association of testosterone with fibrotic progression of NAFLD

	Total testosterone^a^				*P* for trend	1 SD increment for ln (TT)
	Quartile 1	Quartile 2	Quartile 3	Quartile 4		
Men						
Indeterminate group	1.02 (0.46–2.29)	1.23 (0.56–2.75)	0.81 (0.37–1.79)	Ref.	0.697	0.95 (0.68–1.32)
Presence of significant fibrosis	0.35 (0.14–0.90)*	0.65 (0.26–1.67)	0.58 (0.23–1.45)	Ref.	0.044	1.27 (0.87–1.85)
Fibrotic progression of NAFLD	0.45 (0.29–0.72)*	0.66 (0.42–1.05)	0.70 (0.44–1.11)	Ref.	0.001	1.24 (1.07–1.45)*

Data are shown as regression coefficients or odds ratios (95% CI). Multinomial and ordinal logistic regression analyses were used

^a^Quartiles of total testosterone, nmol/L: men: ≤10.75, 10.76–14.06, 14.07–17.88, ≥17.89. Fibrotic progression of NAFLD, ranging from absence of significant fibrosis to indeterminate group and presence of significant fibrosis; NAFLD, nonalcoholic fatty liver disease. The model was adjusted for age, duration of diabetes, current smoking, waist circumference, dyslipidemia, and hypertension. *P < 0.05

### Subgroup analysis

**Fig. 2:**
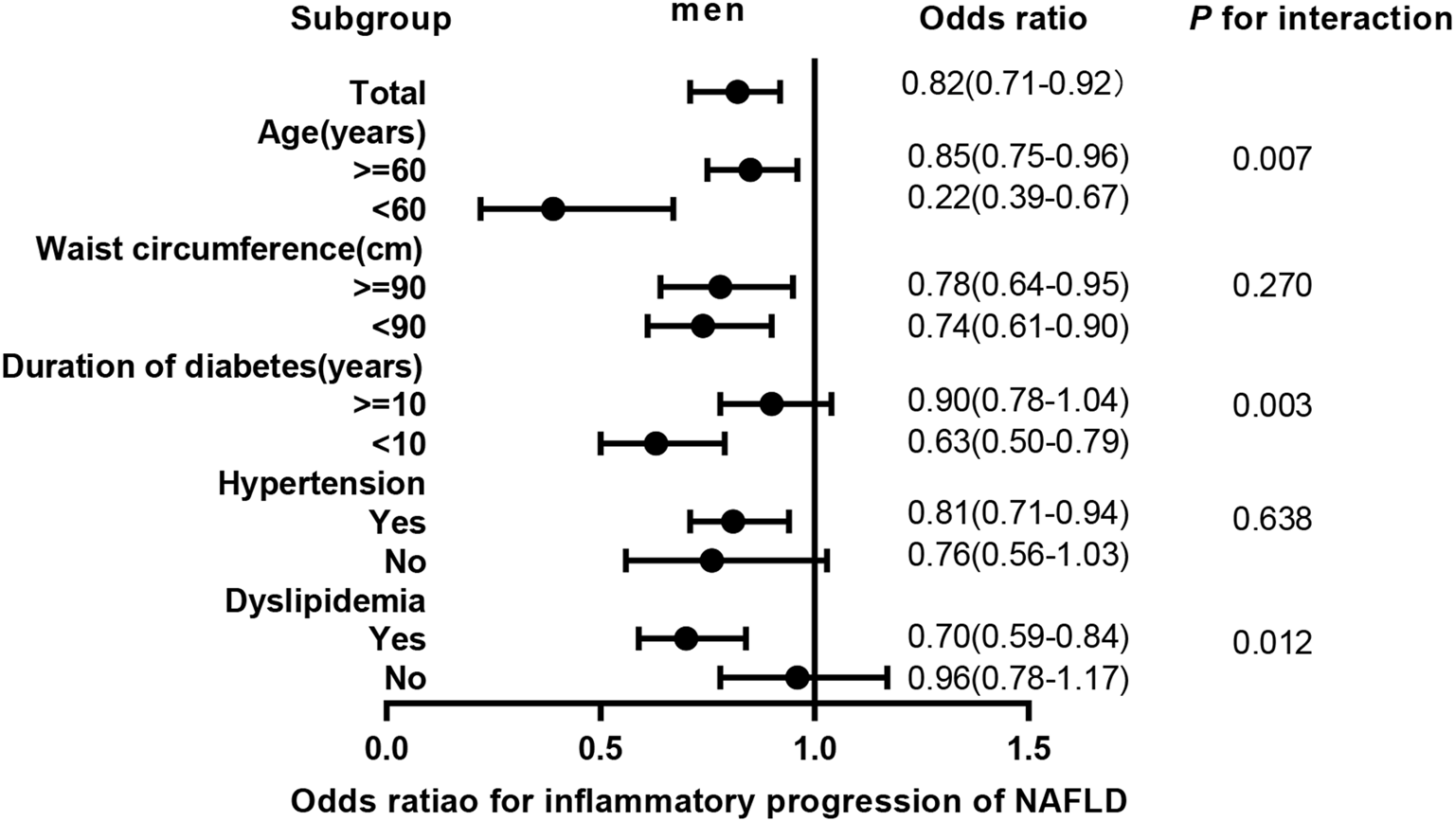
Odds ratios for inflammatory progression of NAFLD. Adjusted ORs for a 1-SD increment of ln (TT) associated with inflammatory progression of NAFLD in the total population and subgroup. Ordinal logistic regression has been used. The ORs with corresponding 95% CIs were adjusted for age, smoking, waist circumference, duration of diabetes, hypertension, and dyslipidemia. TT, Total testosterone; NAFLD, Nonalcoholic fatty liver disease; ORs, Odd ratio; SD, standard deviation; CI, confidence interval

**Fig. 3:**
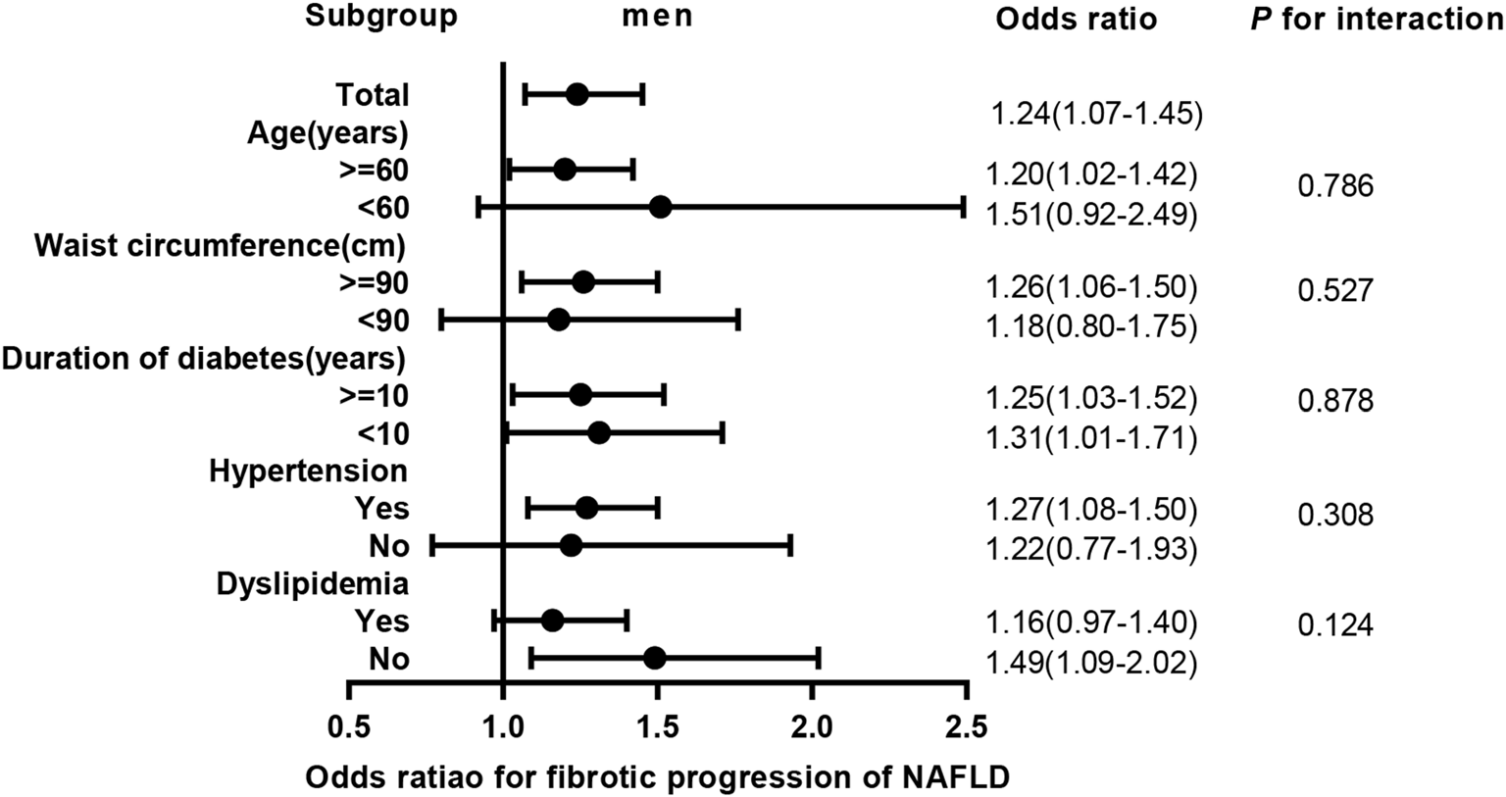
Odds ratios for fibrotic progression of NAFLD. Adjusted ORs for a 1-SD increment of ln (TT) associated with fibrotic progression of NAFLD in the total population and subgroup. Ordinal logistic regression has been used. The ORs with corresponding 95% CIs were adjusted for age, smoking, waist circumference, duration of diabetes, hypertension, and dyslipidemia. TT, total testosterone; NAFLD, nonalcoholic fatty liver disease; ORs, odd ratio; SD, standard deviation; CI, confidence interval

To investigate the association of inflammatory and fibrotic progression of NAFLD with a 1 SD increment of ln (TT) in subgroup of strata variables in Chinese diabetic men, we performed stratified analyses (Figs. 2, 3) We found that the associations of TT and inflammatory progression of NAFLD were significant in age strata (≥60 years or <60 years), waist circumference strata (≥90 cm or <90 cm), duration of diabetes strata (< 10 years), hypertension status (yes), and dyslipidemia status (yes; all *P* < 0.05) and the interaction was significant in age strata, duration of diabetes strata, and dyslipidemia status (*P* for interaction 0.007, 0.003 and 0.012 respectively). As for fibrotic progression, its associations with TT were not significant in age strata (< 60 years), waist circumference strata (< 90 cm), hypertension status (no) and dyslipidemia status (yes; all *P* ≥ 0.05), and the interaction was not significant (all *P* for interaction ≥0.05).

## Discussion

In this population-based study including 1782 male patients with T2DM, we found that TT of male patients was negatively associated with probable NASH and NAFLD inflammatory progression but positively with fibrotic progression. Our results indicate that TT may play significant while opposite roles in inflammatory and fibrotic stages of NAFLD in Chinese type 2 diabetic men.

The association between low level of testosterone and NASH has been found in several studies. Epidemiological studies have reported the negative association between testosterone and liver steatosis or NASH in general male [18, 31, 32]; however, little study has investigated the association in diabetic patients. Diabetic men are predisposed to hypogonadism which is closely associated with metabolic disorders, involving insulin resistance, central obesity, cardiovascular disease, T2DM, and MetS [33]. Our study further revealed that NAFLD in men with T2DM and low testosterone are more likely to develop to NASH, suggesting more attention ought to be paid to testosterone level of men with type 2 diabetes in clinical practice. Testosterone replacement therapy (TRT) has been mainly used to improve symptoms of hypogonadism [34] and several studies demonstrated benefits of TRT to adverse metabolic conditions, such as obesity [35], T2DM, and MetS [36]. A recent prospective study with 40 weeks of follow-up data found testosterone treatment may reduce liver fat in type 2 diabetic men with low level of testosterone [37]. Our study also provide one of possible rationales for TRT in diabetic men with NAFLD. Mechanistically, testosterone may directly or indirectly ameliorate NAFLD inflammation via multifaceted way. Testosterone in male regulates hepatic insulin resistance and steatosis through liver androgen receptors [38]. Castrated rodents have higher concentrations of insulin, hepatic glucose production [39], increased hepatic lipogenesis, decreased fatty acid oxidation, and hepatic lipid export via regulating expression of related gene, such as FAS, AMPK, and MTP [15], which is improved by testosterone supplementation. Androgen receptors combined with testosterone may promote LKB1 expression thereby regulating AMP-activated protein kinase (AMPK)-acetyl-CoA carboxylase (ACC) signaling and reducing lipogenesis in liver [40]. Also, both global and liver-targeted AR-deleted male mice showed abnormal hepatic steatosis and insulin resistance [16, 41]. Increased ectopic lipid in liver contributes to the inflammatory progression of NAFLD by inflammatory factors including IL-6, TNF-α, and adipokines. In addition, testosterone may suppress hepatic inflammation via directly regulating activation of T cells, thus suppressing the secretion of IL-17 [42]. Another potential mechanism lies in endoplasmic reticulum (ER) stress. In castrated rats, testosterone replacement suppressed liver ER stress and activation of JNK and NF-kB, thereby inhibiting hepatic inflammation [43].

Men are predisposed to advanced hepatic fibrosis relative to women. Besides excessive alcohol consumption [44] and higher morbidity of diabetes [45], testosterone may also partly account for this gender difference. However, the relation between testosterone and hepatic fibrosis is still unclear, which is partly due to different measurements of androgens and hepatic fibrosis or limited study population. A cross-sectional study of 98 men reported that higher levels of testosterone were not significantly associated with indices of hepatic fibrosis, including AST-to-Platelet Ratio Index (APRI), fibrosis-4 index (FIB-4), and NAFLD fibrosis score (NFS) [19]. However, in another cross-sectional study of 308 males with hepatitis B in which fibrosis was assessed by FibroSURE-ActiTest, higher TT was associated with increased risk of advanced fibrosis [46]. In the current study with larger sample population, we also found a positive association between testosterone and liver fibrosis in Chinese men with T2DM and NAFLD. Our results indicate that a cautious attitude toward TRT should be taken when NAFLD of diabetic men progresses into fibrosis stage. This positive association can be explained partly by the decrease of liver androgen clearance when there is fibrosis [47]. Additionally, liver androgen receptor can regulate genes involved in cellular differentiation, proliferation, and apoptosis, by which testosterone can influence the fibrotic progression. So, we postulate that the observed higher TT may be the result of damaged liver function and, meanwhile, has an action on the development of fibrosis, thereby reinforcing a vicious circle.

In subgroup analysis, we found that protective effects of TT on inflammatory progression of men with T2DM in age strata (<60 years; OR 0.22, 95% CI 0.39–0.67), duration of diabetes strata (<10 years; OR 0.63 95% CI 0.50–0.79), and dyslipidemia status (yes; OR 0.70 95% CI 0.59–0.84) were relatively more significant (all *P* for interaction <0.05). Meanwhile, we found no interactions between fibrosis progression and testosterone in any strata variables (all *P* for interaction ≥0.05) and the association of TT and fibrosis progression was not significant in NAFLD patient aged <60 years or with dyslipidemia (both *P* ≥ 0.05). Therefore, TRT may have greater advantage for men aged <60 years, with shorter duration of diabetes or with dyslipidemia.

Interestingly, we found that TT increased in aging men with diabetes which is consistent with our previous finding. One potential mechanism is that the testes responsiveness to luteinizing hormone (LH) of Chinese decreased slightly compared with other population. Also, diminished clearance of sex hormone binding globulin (SHBG) may affect the level of TT [48].

Although the current study has some strengths, including the large sample of community-dwelling participants, novelty, and strong quality control, there are also some limitations. First, since it is a cross-sectional study, the causal relationship between TT and NAFLD progression cannot be clarified. Second, we used NFS to assess probable advanced fibrosis in patients with NAFLD and type 2 diabetes and defined probable NASH as concurrence of NAFLD and MetS. Liver biopsy, albeit the gold standard to assess liver inflammation and fibrosis, is an invasive examination, so it could not be used in large-scale epidemiological studies. NFS can predict presence of advanced liver fibrosis with high sensitivity and specificity. Lastly, we did not measure sex hormone binding protein and free testosterone, so we cannot explore the relationship between SHBG or free testosterone and NAFLD progression. However, TT was closely related to free testosterone in the elder [49] and the role of SHBG in severity of NAFLD is debatable [50, 51].

In conclusion, our results indicate that in male patients with T2DM, testosterone may play significant but opposite roles in inflammatory and fibrotic progression of NAFLD. Hence, understanding androgen hormone changes during T2DM and differentiating the stages of NAFLD may help clinicians personalize treatment strategy for male NAFLD patients with T2DM. Further studies are warranted to identify the causality and potential mechanisms of testosterone in NAFLD inflammatory and fibrotic progression.

## Data Availability

The datasets used and/or analyzed during the current study are available from the corresponding author on reasonable request.

## Abbreviations

T2DM: Type 2 diabetes mellitus
NAFLD: Nonalcoholic fatty liver disease
NASH: Nonalcoholic steatohepatitis
MetS: Metabolic syndrome
ORs: Odds ratios
SD: Standard deviation
CI: Confidence interval
TT: Total testosterone
NFS: NAFLD fibrosis score
ALT: Alanine aminotransferase
AST: Aspartate aminotransferase
DBP: Diastolic blood pressure
SBP: Systolic blood pressure
FPG: Fasting plasma glucose
HbA1c: Glycated hemoglobin
HDL-C: High-density lipoprotein
LDL-C: Low-density lipoprotein
SHBG: Sex hormone binding globulin
ER: Endoplasmic reticulum
